# Sequential Anti-PD1 Therapy Following Dendritic Cell Vaccination Improves Survival in a HER2 Mammary Carcinoma Model and Identifies a Critical Role for CD4 T Cells in Mediating the Response

**DOI:** 10.3389/fimmu.2019.01939

**Published:** 2019-08-14

**Authors:** Krithika N. Kodumudi, Ganesan Ramamoorthi, Colin Snyder, Amrita Basu, Yongsheng Jia, Sabrina Awshah, Amber P. Beyer, Doris Wiener, Lian Lam, Hongtao Zhang, Mark I. Greene, Ricardo L. B. Costa, Brian J. Czerniecki

**Affiliations:** ^1^Clinical Science & Immunology Program, H. Lee Moffitt Cancer Center, Tampa, FL, United States; ^2^Department of Breast Oncology, Tianjin Medical University Cancer Institute & Hospital, National Clinical Research Center for Cancer, Tianjin, China; ^3^Perelman School of Medicine, University of Pennsylvania, Philadelphia, PA, United States; ^4^Department of Breast Oncology, H. Lee Moffitt Cancer Center, Tampa, FL, United States

**Keywords:** breast cancer, dendritic cells, PD-1, PD-L1, HER2, immune checkpoints, CD4 T cells, Th1

## Abstract

Patients with metastatic HER2 breast cancer (MBC) often become resistant to HER 2 targeted therapy and have recurrence of disease. The Panacea trial suggested that HER2 MBC patients were more likely to respond to checkpoint therapy if TIL were present or if tumor expressed PD-L1. We assessed whether type I polarized dendritic cells (DC1) could improve checkpoint therapy in a preclinical model of HER2^+^ breast cancer. TUBO bearing mice were vaccinated with either MHC class I or class II HER2 peptide pulsed DC1 (class I or class II HER2-DC1) concurrently or sequentially with administration of anti-PD-1 or anti-PDL1. Infiltration of tumors by immune cells, induction of anti-HER2 immunity and response to therapy was evaluated. Class I or class II HER2-DC1 vaccinated mice generated anti-HER2 CD8 or CD4+ T cell immune responses and demonstrated delayed tumor growth. Combining both MHC class I and II HER2-pulsed DC1 did not further result in inhibition of tumor growth or enhanced survival compared to individual administration. Interestingly class II HER2-DC1 led to both increased CD4 and CD8 T cells in the tumor microenvironment while class I peptides typically resulted in only increased CD8 T cells. Anti-PD-1 but not anti-PD-L1 administered sequentially with class I or class II HER2-DC1 vaccine could improve the efficacy of HER2-DC1 vaccine as measured by tumor growth, survival, infiltration of tumors by T cells and increase in systemic anti-HER2 immune responses. Depletion of CD4+ T cells abrogated the anti-tumor efficacy of combination therapy with class II HER2-DC1 and anti-PD-1, suggesting that tumor regression was CD4 dependent. Since class II HER2-DC1 was as effective as class I, we combined class II HER2-DC1 vaccine with anti-rat neu antibodies and anti-PD-1 therapy. Combination therapy demonstrated further delay in tumor growth, and enhanced survival compared to control mice. In summary, Class II HER2-DC1 drives both a CD4 and CD8 T cell tumor infiltration that leads to increased survival, and in combination with anti-HER2 therapy and checkpoint blockade can improve survival in preclinical models of HER2 positive breast cancer and warrants exploration in patients with HER2 MBC.

## Introduction

Breast cancer is the most commonly diagnosed tumor and a major cause of cancer death among women (1). A subset of breast cancers present expression/amplification of the HER2 protein/oncogene which correlates with increased recurrence rates and poor survival (2–4). HER2-targeted treatments have led to meaningful improvement in clinical outcomes for patients with breast tumors driven by HER2. For example, the HER2-targeted antibodies, trastuzumab and pertuzumab, combined with docetaxel improved the median overall survival (mOS) of patients with HER2-positive (HER2^+^) metastatic breast cancer (MBC) to 56.5 months compared to 20.3 months for patients receiving chemotherapy alone (5, 6). In the second line setting, treatment with the antibody drug conjugate ado-trastuzumab (T-DM1) improved the mOS in patients with trastuzumab-resistant HER2^+^ MBC from 15.9–25.9 to 22.7–29.9 months when compared to chemotherapy or to treatment with small tyrosine kinase inhibitor (lapatinib) (7). Taken together these data support the clinical validity of the HER2 antigen as a valid predictive biomarker of clinical benefit for treatment with HER2-targeted therapies even after disease progression with approved targeted agents. Notwithstanding the recent advances, HER2+ MBC will eventually acquire resistance to HER2-targeted therapies and disease progression will ensue. Therefore, alternative or other combinatorial approaches are needed to overcome resistance to HER2-targeted treatment and improve clinical outcomes.

The presence of tumor infiltrating lymphocyte (TIL) in HER2^+^ breast cancer is consistently associated with improved prognosis and better survival (8–10). Trastuzumab treatment of breast cancer patients with the presence of TIL have improved survival and complete response to neoadjuvant therapy (9–12). HER2 antibody treatment has been reported in preclinical studies to induce adaptive and innate immune responses and to increase infiltration of immune cells into the tumor microenvironment (13).

Several co-inhibitory immune checkpoint signals such as programmed death 1 (PD-1) receptor/PD-ligand 1 (PD-L1) have been shown to inhibit anti-tumor immune responses (14, 15). Binding of PD-L1 to its receptor PD-1 on the surface of T cells can induce TIL exhaustion and evade anti-tumor immunity (16). Preclinical studies combining antibodies against PD-1 improved the immune-mediated effects of anti-HER2 monoclonal antibody therapy (17). These data provide a strong rationale for the use of immune checkpoint inhibitors as a combinatorial approach in HER2^+^ breast cancer. The phase 1b/2 KEYNOTE-014/PANACEA trial evaluated the efficacy of pembrolizumab (anti-PD-1 antibody) in combination with trastuzumab in HER2^+^ MBC patients that progressed after previous HER2 targeted therapies. Fifteen percent of the patients that were PD-L1 positive achieved an overall response and no overall response was observed in the PD-L1 negative cohort (18). Another study in a phase 1 trial evaluated trastuzumab in combination with durvalumab (anti-PD-L1) in metastatic HER2^+^ breast cancer patients and no impact on objective responses was observed and all the patients enrolled in the trials had lower than 1% PD-L1 expression (19). Overall, these studies suggest that checkpoint inhibitors combined with anti-HER2 therapy have minimal impact. Developing strategies that can increase T cell infiltration in tumors may improve the efficacy of these therapies.

Vaccine strategies using dendritic cells to activate the immune system and generate Th1 immune responses have been extensively studied (20, 21). It has been well-documented that Th1 cytokine, IFN-γ can induce PD-L1 expression on tumor cells (22). In preclinical models of various cancer types with increased levels of immune checkpoint molecules expression on TIL, immune checkpoint blockade in combination with vaccine strategies has shown a superior response compared to monotherapy (23, 24). The role of CD8^+^ T cells to generate anti-tumor immunity in HER2^+^ breast cancers has been shown in various clinical trials (25) and has had minimal clinical impact (26, 27). However, the role and prognostic value of CD4^+^ T cells has not been extensively studied in breast cancer. Previous findings from our lab have shown that anti-HER2 CD4^+^ T helper cell (Th1) immunity plays a crucial role in cancer therapy and peripheral loss of the Th1 response correlates with poor treatment response and prognosis (28). Administration of class II HER2 peptide-pulsed Type I polarized dendritic cell (DC1) vaccine induced a strong anti-HER2 immune response with pathologic complete response rate (pCR) in HER2^+^ DCIS patients (29–31). Very little is known about the role of anti-HER2 CD4^+^ Th1 immune responses in combination with immune checkpoint therapy. Based on these preliminary findings, we hypothesized that HER2 peptide pulsed DC1 vaccine could prime an anti-HER2 response and generate anti-HER2 Th1 immune responses leading to the conversion of “cold” to “hot” tumors and thus improve the efficiency of immune checkpoint antibody therapy. The goal of this study was to investigate the anti-tumor efficacy of HER2 peptide pulsed DC1 vaccine in combination with PD-1/PD-L1 blockade and HER2 targeted therapy in a preclinical model of HER2^+^ breast cancer.

## Materials and Methods

### Animals

This study was carried out in strict accordance with the recommendations in the Guide for the Care and Use of Laboratory Animals of the National Institutes of Health. The protocol was reviewed and approved by the Institutional Animal Care and Use Committee at the University of South Florida (#A4100-01). Mice were euthanized by CO_2_ inhalation according to the American Veterinary Medical Association Guidelines. Mice were observed daily and euthanized if a solitary subcutaneous tumor exceeded the end point. All efforts were made to minimize suffering. Female Balb/C mice (6–8 weeks old) were purchased from Charles river. Mice were housed at the Animal Research Facility of the H. Lee Moffitt Cancer Center and Research Institute.

### Tumor Cell Lines

TUBO breast cancer cell line (kind gift from Dr. Wei Zen Wei, Wayne State University) was cloned from a spontaneous mammary tumor in BALB/c mice transgenic for the rat Her-2/neu gene (BALB-neuT) (32) and was maintained by serial *in vitro* passages in complete medium (CM). Complete media consisted of RPMI 1640 (Fisher Scientific, Cat. No. MT-10-040-CM) supplemented with 10% heat-inactivated FBS (Fisher Scientific, Cat. No. MT35010CV), 0.1 mM nonessential amino acids (Fisher Scientific, Cat. No. 25025CI), 1 mM sodium pyruvate (Fisher Scientific, Cat. No. 25000CI), 2 mM fresh L-glutamine (Fisher Scientific, Cat. No. 25005CI), 100 mg/ml streptomycin and 100 U/mL penicillin (Fisher Scientific, Cat. No. MT-30-002-CI), 50 mg/mL gentamicin (Gibco, Cat. No. 15750060), 0.5 mg/mL fungizone (Gibco, Cat. No. 15290018) (all purchased from Life Technologies, Rockville, MD), and 0.05 mM 2-ME (Gibco, Cat. No. 21985023).

### DC Generation

Bone marrow (BM) cells were harvested from femurs and tibias of Balb/C mice as described previously (33). Briefly, BM cells were flushed into a cell suspension in RPMI 1640, and RBCs were lysed using ACK lysing buffer. Cells were cultured with rFLT3L (VWR Peprotech, Cat. No. 10778-670) at 25 ng/mL and rmIL-6 (R&D Systems, Cat. No. 406-ML-025) at 30 ng/mL in T75 flasks and incubated for 6 days at 37°C and 5% CO2. The BM cells were then harvested, washed with RPMI 1640 and cultured with 50 ng/mL of rmGM-CSF (R&D Systems, Cat. No. 415-ML-050) and 10 ng/mL of rmIL-4 (R&D Systems, Cat. No. 404-ML-050) overnight, followed by DC1 maturation for 6–8 hours (h) with DC1 polarizing signals: CPG/ODN1826 (InVivoGen, Cat. No. tlrl-1826), a TLR 9 agonist at 10 ng/mL and lipopolysaccharide (LPS) (Millipore Sigma, Cat. No. L4391), a TLR-4 agonist at 20 ng/mL as described previously (33). When used for vaccination, DC1 cells were pulsed with multi-epitope peptides from the rat HER2/neu (rHER2/neu) oncogene at the concentration of 10 μg/ml of each peptide individually overnight; p5 (ELAAWCRWGFLLALLPPGIAG), p435 (IRGRILHDGAYSLTLQGLGIH), and p1209 (SPPHPSPAFSPAFDNLYYWDQ) and were pooled for class II HER2-DC1 vaccine studies (34). DC1 were pulsed with class I rat HER2/neu peptide p66 (TYVPANASL) for class I HER2-DC1 vaccine studies (35). All the peptides were synthesized from Bachem Americas, Inc. DC maturation was confirmed in a subset of samples at 24 h post addition of LPS and CPG by FACS analysis of cell surface markers, MHC class II (I Ad), CD80, CD86, and CD40 (FITC anti-mouse I-Ad (Clone 39-10-8, Biolegend, Cat. No. 115006); PE anti-mouse CD80 (Clone 16-10A1, Biolegend, Cat. No. 104708) anti-mouse CD40; PE anti-mouse CD86 (Clone GL-1, Biolegend, Cat. No. 105008); PE anti-mouse CD40 (Clone 3/23, Biolegend, Cat. No. 124610). IL-12 (p70) secretion by DC1 in culture supernatants was measured by standard IL-12 (p70) ELISA from R& D systems (Cat. No. M1270).

### Monoclonal Antibodies

The monoclonal antibodies anti-PD-1 (clone RMP1-14, Cat. No. BE0146) and anti-PDL-1 (clone 10F.9G2, Cat. No. BE0101) were purchased from BioXCell (West Lebanon, NH). InVivoMAb rat IgG2a isotype (BioXCell, Cat. No. BE0089) was used as control. Anti-HER2 mouse monoclonal antibody 7.9.5 was a kind gift from Dr. Mark Greene, University of Pennsylvania and clone 7.16.4 was purchased from BioXCell (Cat. No. BE0277).

### Immunofluorescence Staining for HER2

TUBO cells were grown to 80% confluence on sterile round glass coverslips in a six well tissue culture plate. Cells were washed three times with PBS and fixed in 4% (wt/vol) paraformaldehyde (Fisher Scientific, Cat. No. 50-980-487) for 15 min. Next, cells were permeabilized with 0.2% Triton X (Sigma Aldrich, Cat. No. T8787) for 10 min and washed three times with PBS. After washing, cells attached to cover slips were incubated with 5% (wt/vol) bovine serum albumin (BSA) (Fisher Scientific, Cat. No. BP1605) in PBS for 1 h at room temperature. Cells were then incubated with monoclonal primary anti-HER2/ErbB2 antibody (Cell Signaling Technology, Beverly, MA, Cat. No. 2165) over night at 4°C followed by incubation with Alexa Fluor 594-conjugated anti-rabbit secondary antibody (Cell Signaling Technology, Beverly, MA, Cat. No. 8889) for 1 h at room temperature in the dark. After being washed with PBS three times, coverslips were mounted using VECTASHIELD Antifade Mounting Medium with DAPI (Vector Laboratories, Cat. No. H-1200). The stained coverslips were examined and imaged using a Zeiss Apotome.2 fluorescence microscope (Carl Zeiss Inc., Thornwood, NY).

### Western Blot Analysis

Total protein was isolated from TUBO and 4T1 cells for Western blot analysis. Briefly, cells were lysed with 1X RIPA buffer (EMD Millipore™, Cat. No. 20-188) containing protease inhibitor (Millipore Sigma, Cat. No. P8340) and phosphatase inhibitor (ThermoScientific Pierce, Cat. No. A32957), for 20 min at 4°C. The cell lysate was centrifuged at 15,000 rpm for 20 min and the supernatant containing total protein was collected and stored at −80°C until further use. Protein concentration was measured by Bradford protein assay (Bio-Rad, Hercules, CA, Cta. No. 5000006). For Western blotting, 20 μg of each protein sample was resolved in a 4–12% SDS-PAGE and then transferred to a polyvinylidene difluoride (PVDF) membrane (Millipore Sigma, Cat. No. IPVH00010) using eBlot® L1 wet transfer system (GenScript, Piscataway, NJ). Membranes were blocked with 5% bovine serum albumin (BSA)/TBS-T for 1 h at room temperature, followed by overnight incubation with primary monoclonal anti-HER2/ErbB2 antibody (Cell Signaling Technology®, Cat. No. 2165) (1:1,000 dilution in 5% BSA/TBS-T) at 4°C. The next day, membranes were washed three times, 10 min each wash with TBS-T, and was probed with goat anti-rabbit IgG (H + L)-HRP conjugated secondary antibody (Cell Signaling Technology®, Cat. No. 7074; 1:5,000 dilution in 5% non-fat dry milk/TBS-T) for 1 h at room temperature and detected using the ECL western blotting detection system (Thermo Scientific Pierce, Cat. No. 32106). β-actin was used as endogenous control for all Western blot data analyses.

### *In vivo* Treatments

A total of 2.5 × 10^5^ TUBO cells were injected subcutaneously (s.c.) in female Balb/C mice. Seven days later when tumors were palpable, mice were treated with six doses of class I or class II HER2-DC1 vaccine subcutaneously either once, twice or three times weekly. For combination therapy, mice received either class I or class II HER2-DC1 vaccine (1 × 10^6^ cells/mouse/subcutaneous injection/ 100 μl) with 150 μg of monoclonal antibody (isotype control or anti-PD-1 or anti-PD-L1) intraperitoneally twice a week concurrently or DC1 vaccination given first, followed by checkpoint antibodies. Mice continued to receive checkpoint antibody treatment twice a week until the tumor reached a size of 2 cm in diameter. Tumor size was measured and recorded every 2–3 days. Six mice per group were used and each experiment was performed three times. For functional analysis, mice were euthanatized at day 28 after tumor injection. Tumors and splenocytes were harvested for *in vitro* assays.

### CD4 T Cells Depletion

Anti-CD4 antibody (InVivoMab clone GK1.5 purchased from BioXCell, Cat. No. BE0003-1) was used to deplete CD4 T cells in the experimental mice. Three days before the TUBO injection, Balb/c mice were administered intraperitoneally with 300 μg of anti-CD4 antibody and continued with two injections per week until the end point. When tumors were palpable around days 7–10, mice were treated with multi-epitope class II HER2-DC1 vaccine subcutaneously twice a week. Another group of TUBO bearing mice without CD4 depletion received class II HER2-DC1 vaccine twice a week for total of six doses. Mice treated with or without CD4 depleting antibody were randomized into two groups to receive a follow up treatment with anti-PD1 antibody twice a week until the end point. Tumor size was measured and recorded twice a week.

### HER2 Blockade in Combination With Class II HER2-DC1 and Anti-PD-1 Therapy

For *in vivo* treatments of HER2 targeted therapy in combination with HER2-DC1 vaccine and anti-PD-1 antibody, Balb/C mice were injected with 3 × 10^4^ TUBO cells/50 μl in mammary fat pad per mouse. On day 12 after TUBO cells injection and when tumors were palpable, mice were randomized in four groups: (1) untreated, (2) anti-PD-1 therapy, (3) class II HER2-DC1 vaccine, and (4) combination therapy with anti-HER2, anti-PD-1, and HER2-DC1. For combination treatments, mice received anti-HER2 antibodies (clone 7.16.4 and 7.6.5) (50 μg/clone/mouse) on day 12. One week after anti-HER2 antibody treatment, mice received HER2-DC1 vaccine subcutaneously twice a week concurrently with combined 7.16.4 and 7.6.5 antibodies given once a week for 3 weeks. Upon completion of the combination treatment, follow up with anti-PD-1 antibody was given twice a week until the end point. Tumor growth was monitored twice a week and tumor volume was calculated following the formula: (L x W^2^)/2 = mm^3^.

### Flow Cytometry

On day 28 after tumor injection, spleens and/or tumors were harvested under sterile conditions. Single-cell suspensions were prepared, and red blood cells were lysed using ACK lysis buffer. Tumor cell suspensions were prepared from solid tumors by enzymatic digestion in HBSS (Fisher Scientific, Cat. No. MT-21-022-CM) containing 1 mg/ml collagenase (Cat. No. C9891 and C-5138), 0.1 mg/ml DNase I (Cat. No. DN25), and 2.5 U/ml of hyaluronidase (Cat. No. H-6254-1G) (all purchased from Millipore Sigma) with constant stirring for 2 h at room temperature as described previously (36). For analysis of immune cell populations, 1 × 10^6^ cells (tumor digest suspension) were incubated for 30 min with Live/ Dead Zombie near IR (Biolegend, Cat. No. 423106) for 30 min in 1X PBS at room temperature in dark. After washing cells with 1X PBS, cells were stained with anti-mouse CD3 Alexa 488 (Clone 17A2, Biolegend, Cat. No. 100210), anti-mouse CD4 BV805 (Clone GK1.5, BD Biosciences, Cat. No. 564922), and anti-mouse CD8 pacific Blue (Clone 53–6.7, BD Bioscience, Cat No. 558106) and anti-mouse PD-1 BV605 (Clone 29F.1.A12, Biolegend, Cat. No. 135220) for 20 min on ice in staining buffer for surface expression analysis, according to the manufacturer's instructions (all antibodies were purchased from BD Biosciences). Samples were analyzed using an LSRII (BD Biosciences) cytometer and FACS data was analyzed using FlowJo software (Tree Star).

### Functional Assays

To examine antigen specificity following HER2-DC1 vaccination in TUBO bearing mice, 2 × 10^6^ splenocytes from control and treatment groups were cultured with 2 μg/ml of class I (p66) peptide, control peptide, or no peptide (complete media only) or multi-epitope class II rat HER2/neu peptides (p5, p435, p1209) individually for 3–4 days. Culture supernatants were collected to measure IFN-γ secretion using a standard quantikine IFN-γ ELISA (R&D systems, Cat. No. SMIF00) according to manufacturer's recommendations.

### Statistical Analysis

The Mann–Whitney test (unpaired) or the Student's *t-*test was used to compare results between two treatment groups. All statistical analyses of data were performed using GraphPad Prism software. Statistical significance was achieved at *p* < 0.05.

## Results

### DC1 From Balb/C Mice Secrete IL-12 and Express CD80, CD86, and CD40

**Figure 1:**
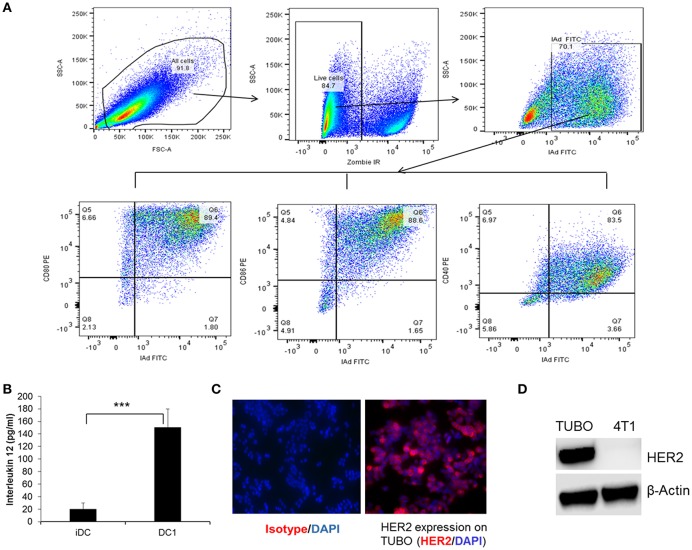
Immunophenotyping and function of type I polarized DC1 from Balb/C mice. **(A)** DC collected after maturation with CpG and LPS were stained for MHC class II (IAd), CD80, CD86, and CD40 and data were acquired on LSRII flow cytometer and analyzed by FlowJo software. Flow gating strategy and representative flow dot plot of DC1 staining for MHC class II, CD80, CD86, and CD40. **(B)** Culture supernatants were collected before and after DC maturation and measured for IL-12 secretion using standard ELISA. **(C)** HER2 expression on TUBO cells using immunofluorescence staining. **(D)** Western blot analysis of HER2 protein expression on tumor cells. *P*-values were determined by Student *t*-test. ***p < 0.001.

To examine the phenotype and the maturation status of DC generation from bone marrow of Balb/C mice, DC1 were collected following maturation and stained for the expression of cell surface markers, class II (I-Ad), CD80, CD86, and CD40 and data was acquired on flow cytometer as described in the Materials and Methods section. Functional status of DC1 was measured by IL-12 production 24 h after addition of final maturation signals. Culture supernatants from immature DC (iDC) were used as control. Flow gating strategy is shown in Figure 1A. Cells were gated on live population followed by gating on MHC class II (IAd) positive cells for CD80, CD86, and CD40 expression. Addition of CPG and LPS resulted in a higher percentage of DC maturation surface markers CD80, CD86, and CD40 (Figure 1A) with higher levels of IL-12 production compared to iDC (Figure 1B, *p* < 0.001). To examine the anti-tumor efficacy of DC1 vaccine in a HER2^+^ breast tumor model, we utilized the TUBO cell line which was derived from a spontaneous mammary tumor in Balb/c mice transgenic for the rat Her-2/neu gene (BALB-neuT). As shown in Figure 1C, we confirmed the surface expression of HER2 on TUBO cells by immunofluorescence. HER2 protein expression was also confirmed by western blot, along with 4T1, a triple negative cell line (negative for ER, PR, and HER2), as negative control (Figure 1D). Overall, our data suggests that DC1 we generated from bone marrow of Balb/C mice secrete high levels of IL-12, and express mature DC phenotype.

### Class I or Class II HER2 Peptide- DC1 Vaccine Delay Tumor Growth and Induce Anti-HER2 Th1 Immune Response With Increased T Cell Infiltration in TUBO Bearing Mice

**Figure 2:**
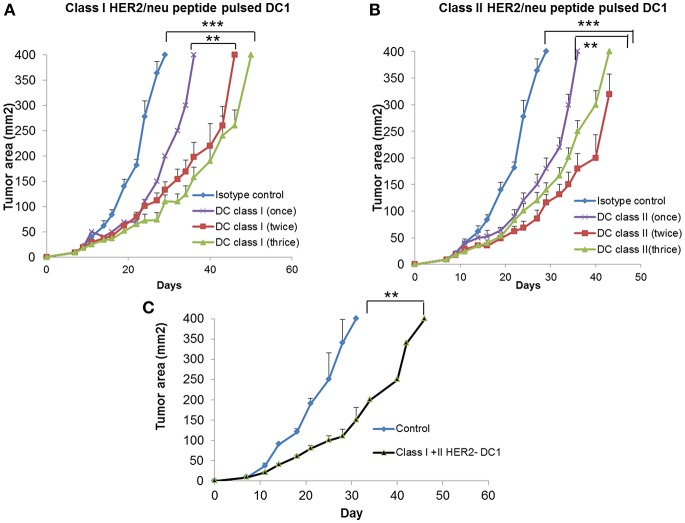
Vaccination with DC1 pulsed with class I or class II HER2 peptides delays tumor growth in TUBO bearing mice. **(A)** DC1 was generated as described in methods section and pulsed with class I rHER2 peptide p66 (class I HER2-DC1). Balb/C mice were injected with 2.5 × 10^5^ TUBO cells subcutaneously on the right flank on day 0. When tumors were palpable on day 7, mice received class I HER2-DC1 vaccine subcutaneously on the left flank once, twice or three times a week for a total of six doses. *N* = 8 mice/group was used for these studies. Tumor area was measured 2–3 days a week. **(B)** Balb/C mice received tumor cells and HER2-DC1 vaccine as described above. DC1 were pulsed with 10 μg/ml of each peptides from the rat HER2 (rHER2) oncogene; p5 (ELAAWCRWGFLLALLPPGIAG), p435 (IRGRILHDGAYSLTLQGLGIH), and p1209 (SPPHPSPAFSPAFDNLYYWDQ) and p66 (TYVPANASL). **(C)** Balb/C mice received tumor cells as described above and received both class I and class II pulsed HER2-DC1 vaccine. Data shown are the representative from three independent experiments and are shown as mean number ± SEM ***p < 0.001, **p < 0.01 using Student *t*-test.

To examine the anti-tumor efficacy of DC1 vaccine, we utilized the HER2 positive TUBO model. Balb/C mice were injected with TUBO cells (2.5 × 10^5^ cells/mouse/s.c.,) on day 0. Starting on day 7 when tumors were palpable, TUBO bearing mice were treated with either class I HER2 pulsed DC1 vaccine (Class I HER2-DC1, 1 × 10^6^ DC1/mouse/100 μl /s.c.,) or class II HER2 peptide pulsed DC1 (Class II HER2-DC1). Treatment groups included TUBO bearing mice with no treatment, HER2-DC1 vaccine given once a week, twice or three times a week for total of up to six injections. TUBO bearing mice receiving class I or class II pulsed HER2-DC1 vaccine showed significantly delayed tumor growth compared to control mice (Figures 2A,B; *p* < 0.001) irrespective of whether HER2-DC1 vaccine was given once, twice or three times weekly. However, TUBO bearing mice receiving HER2-DC1 vaccine twice or three times a week had reduced tumor burden compared to the mice receiving weekly dose of class I or class II HER2-DC1 vaccine (Figures 2A,B). Although there was a significant delay in tumor growth in mice receiving HER2-DC1 vaccine given three times a week, as shown in Figure 2A, toxicity was observed (weight loss, hunched and sudden death) in this group. There was none observed in mice receiving once or twice weekly class I HER2-DC1 vaccine.

Since we observed anti-tumor effects of class I and class II HER2-DC1 vaccines shown in Figures 2A,B, we examined whether combining both class I and class II pulsed HER2-DC1 vaccine could have a synergistic effect in delaying tumor growth. As shown in Figure 2C, combined class I and class II pulsed HER2-DC1 vaccine given twice a week significantly delayed tumor growth in TUBO bearing mice but there was no additive benefit in reducing tumor burden (Figure 2C) compared to class I or class II HER2-DC1 alone as shown in Figures 2A,B.

### HER2-DC1 Vaccine Generates Anti-HER2 Th1 Immune Responses in TUBO Bearing Mice

**Figure 3:**
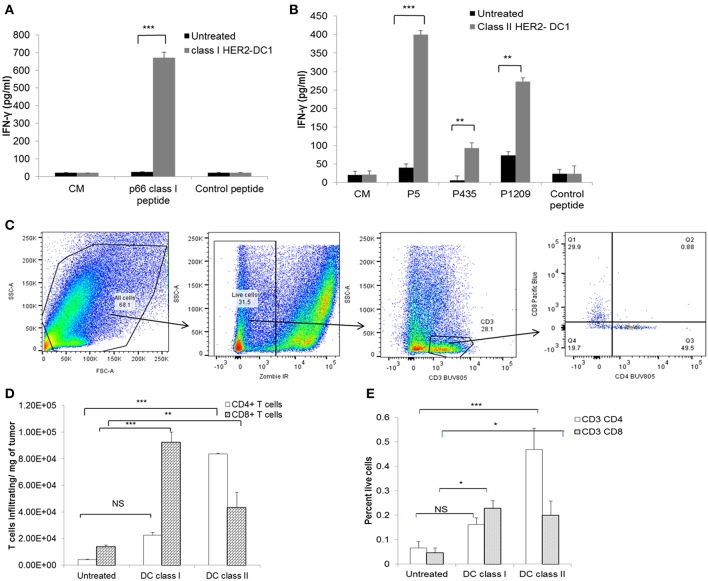
Vaccination with HER2-DC1 enhances anti-HER2 Th1 immune response and increases T cell infiltration in TUBO bearing mice. **(A)** Splenocytes from class I HER2-DC1 vaccinated mice were re-stimulated with p66 rHER2 peptide. Culture supernatants were collected after 3 days and IFN-γ secretion was measured by standard IFN-γ ELISA. **(B)** Splenocytes from class II HER2-DC1 vaccinated mice were re-stimulated with (class II) rat HER2 peptides (rHER2); p5 (ELAAWCRWGFLLALLPPGIAG), p435 (IRGRILHDGAYSLTLQGLGIH), and p1209 (SPPHPSPAFSPAFDNLYYWDQ) individually and IFN-γ secretion was measured by standard IFN-γ ELISA. **(C)** Flow gating strategy and analysis of T cell infiltration in tumors by flow cytometry. Tumors were harvested on day 30 and processed as described in Materials and Methods section. Single cell suspension was stained for live/dead near IR, CD3, CD4, and CD8. Data were acquired on an LSRII flow cytometer and analyzed by FlowJo software. **(D)** Bar graphs represent T cell infiltration gated on live cells per mg of tumor and **(E)** percent T cells of all live cells within the tumor single cell suspension;. ***p < 0.001, **p < 0.01, *p < 0.05 using Student *t*-test.

Next, we evaluated whether vaccination with HER2-DC1 could generate strong anti-HER2 Th1 immune responses in TUBO bearing mice. Spleens were harvested 1 week after the last DC1 vaccination and splenocytes were cultured with class I or class II peptides as described in the Materials and Methods section. Re-stimulation of splenocytes from class I HER2-DC1 vaccinated mice with p66 (class I) peptide had significantly increased IFN-γ secretion compared to splenocytes from untreated mice (Figure 3A, *p* < 0.001). Similarly, re-stimulation of splenocytes from the class II HER2 peptide pulsed DC1 group had higher levels of IFN-γ production in response to HER2 peptides, p5, p1209, and p435 peptides (Figure 3B, *p* < 0.001). These findings suggest that class I and class II HER2-DC1 vaccine can generate anti-HER2 CD8^+^ and CD4^+^ Th1 specific immune responses and can delay tumor growth in HER2^+^ TUBO bearing mice. We next examined whether vaccination with HER2-DC1 vaccine could improve T cell infiltration within the tumor. Tumors were excised from control and treatment groups and single cell suspensions were prepared and stained for cell surface markers (Live/Dead Zombie near IR, CD3, CD4, and CD8 gated on live cells) as described in Materials and Methods section. The flow gating strategy to identify CD4^+^ and CD8^+^ tumor infiltrating lymphocytes is shown in Figure 3C. Class I (p66) HER2-DC1 vaccine in TUBO bearing mice led to a significant increase in tumor infiltrating CD8^+^ T cells but not CD4^+^ T cells, while class II HER2-DC1 vaccine significantly increased both CD4 and CD8^+^ T cell infiltration compared to untreated controls (Figures 3D,E, *p* < 0.001). Figure 3D represents the T cell infiltration per milligram of tumor and Figure 3E shows percent of T cells of all live cells from tumor digest suspension. This data suggests that HER2-DC1 vaccine enhances T cell infiltration within the tumor in TUBO bearing mice.

### TIL From HER2-DC1 Vaccinated Mice Express Higher Levels of PD-1 Checkpoint Receptor

**Figure 4:**
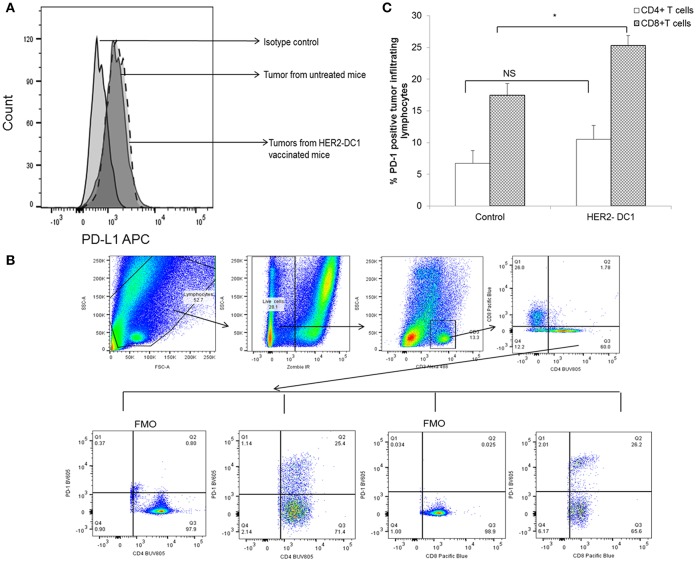
PD-1 expression on TIL and PD-L1 expression on tumors of TUBO bearing mice. **(A)** Tumors were harvested 1 week after the last HER2-DC1 treatment and single suspensions were prepared as described in Materials and Methods section. Single cell tumor digest suspensions were stained for PD-L1 expression and data were acquired on a LSR-II and analyzed using Flowjo software. **(B)** Flow gating strategy of PD-1 expression on tumor infiltrating T cells. **(C)** Tumors were harvested as described in Materials and Methods section and flow staining was performed. Bar graphs show the percent PD-1 expression on CD4 and CD8+ TIL *p < 0.05, NS, Not significant using Student *t*-test.

Inhibitory receptors such as PD-1 expressed on T cells and their ligands such as PD-L1 expressed on tumor cells have been shown to contribute to immune mediated suppression. We investigated whether HER2-DC1 has any effect in modulating the expression of PD-L1 on tumor cells and PD-1 receptor expression on TIL. Tumors were harvested from experimental mice, a single cell suspension was prepared and flow staining was performed as described in Materials and Methods section. As shown in Figure 4A, PD-L1 expression was observed in tumors from untreated mice and HER2-DC1 vaccinated tumor-bearing mice. We did not observe any difference in the expression levels of PD-L1 between tumors from control and HER2-DC1 vaccinated mice. We evaluated the expression of PD-1 on tumor infiltrating lymphocytes. Flow gating strategy is shown in Figure 4B. Cells were gated on live population followed by gating on CD3 positive cells. PD-1 expression on CD4 and CD8 cells was analyzed on samples by gating on Fluorescent minus one (FMO) controls. We observed increased PD-1 expression on CD8^+^ T cells infiltrating within the tumor following HER2-DC1 vaccination compared to control mice (Figure 4C; *p* < 0.05). In contrast to CD8^+^ T cells, there was only a modest but not statistically significant increase in PD-1 expression on CD4^+^ T cells within the tumor following HER2-DC1 vaccination compared to untreated control (Figure 4C). This data suggests that blockade of immune checkpoints in combination with HER2-DC1 vaccine may improve the anti-tumor immune responses in the preclinical model of HER2 positive TUBO breast cancer.

### Class I HER2-DC1 Vaccine in Combination PD-1/PD-L1 Blockade

**Figure 5:**
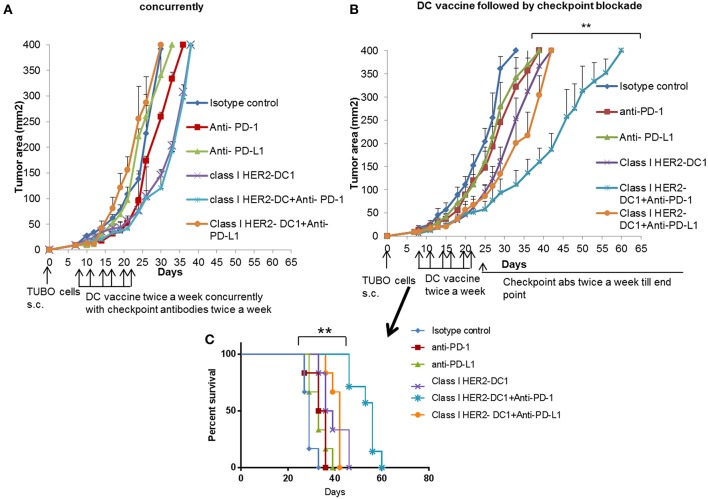
Optimal scheduling of checkpoint antibodies is critical in improving the efficacy of class I HER2-DC1 vaccine in TUBO bearing mice. **(A)** Balb/C mice were injected with 2.5 × 10^5^ TUBO cells subcutaneously on the right flank on day 0. When tumors were palpable on day 7, mice received class I HER2-DC1 vaccine subcutaneously on the left flank twice a week for a total of six doses concurrently with intraperitoneal injection of 150 μg/mouse/200 μl of anti-PD-1 or anti-PD-L1 antibody. **(B)** Balb/C mice were injected with 2.5 × 10^5^ TUBO cells subcutaneously on the right flank on day 0. When tumors were palpable on day 7, mice received class I HER2-DC1 vaccine (1 × 10^6^ DC1/mouse/100 μl) subcutaneously on the left flank twice a week for a total of six doses. Anti-PD-1 or anti-PD-L1 antibody therapy (150 mg/200 ml/mouse/intraperitoneally) twice a week was started after the last injection of HER2-DC1 and continued until the end point. *N* = 8 mice/group was used for these studies and the line graph shown is the representative of triplicate experiments. **(C)** Survival curve. Data shown are the representative from three independent experiments and are shown as the mean number ± SEM. *P*-values were determined by unpaired student *t*-test **(A,B)** or a log-rank test **(C)**. **p < 0.01.

The presence of TILs has been associated with a favorable prognosis in HER2^+^ breast cancer and to potentially predict responders to immune checkpoint blockade (37, 38). Targeting the PD-1 pathway with pembrolizumab in combination with trastuzumab has shown efficacy in HER2 positive trastuzumab resistant patients. The overall response was encouraging in PD-L1 positive cohort. However, there was no overall response in the PD-L1 negative cohort (18). Since we observed increased PD-1 expression on TILs and PD-L1 expression on tumors cells following HER2-DC1 vaccination in TUBO bearing mice, we investigated whether blockade of immune checkpoints, PD-1 or PD-L1 in combination with HER2-DC1 vaccine would enhance the anti-tumor immune response in TUBO bearing mice. Balb/C mice were injected with TUBO cells on day 0. On day 7, when tumors were palpable, two different treatment regimens were followed to examine the efficacy of combination therapy. One group of mice received checkpoint monoclonal antibodies (anti-PD-1 or anti-PD-L1) in combination with class I HER2-DC1 concurrently. Another group of mice received class I HER2-DC1 vaccine twice a week and at the completion of sixth HER2-DC1 vaccine, mice received anti-PD-1 or anti-PD-L1 antibody therapy twice a week until the end point. In addition other treatment groups received either HER2-DC1 alone, anti-PD-1, or anti-PD-L1 antibody as monotherapy. Control mice received isotype control antibody as described in Materials and Methods section. As shown in Figure 5A, TUBO- bearing mice that received Class I (p66) HER2 peptide pulsed DC1 concurrently with intraperitoneal injection of anti-PD-1 or anti-PD-L1 monoclonal antibodies had no significant delay in the tumor growth compared to mice treated with class I (p66) HER2-DC1 alone. However, TUBO bearing mice that received Class I (p66) HER2 peptide pulsed DC1 vaccine followed by treatment with anti-PD-1 monoclonal checkpoint antibodies had a significant delay in tumor growth compared to the mice that received DC1 or checkpoint antibodies alone (Figure 5B). Importantly, TUBO bearing mice that received Class I (p66) HER2 peptide pulsed DC1 in combination with anti-PD-1 antibody had significant delay in tumor growth and doubled the survival rate in TUBO bearing mice, compared to mice that received single treatment or no treatment (Figures 5B,C, *p* < 0.01). However, sequential combination of Class I (p66) HER2-DC1 with anti-PD-L1 did not have an impact on delaying tumor growth or survival benefit compared to DC1 alone (Figures 5B,C).

### Class II HER2 Peptides Pulsed DC1 in Combination With Anti-PD-1 Antibody Therapy

**Figure 6:**
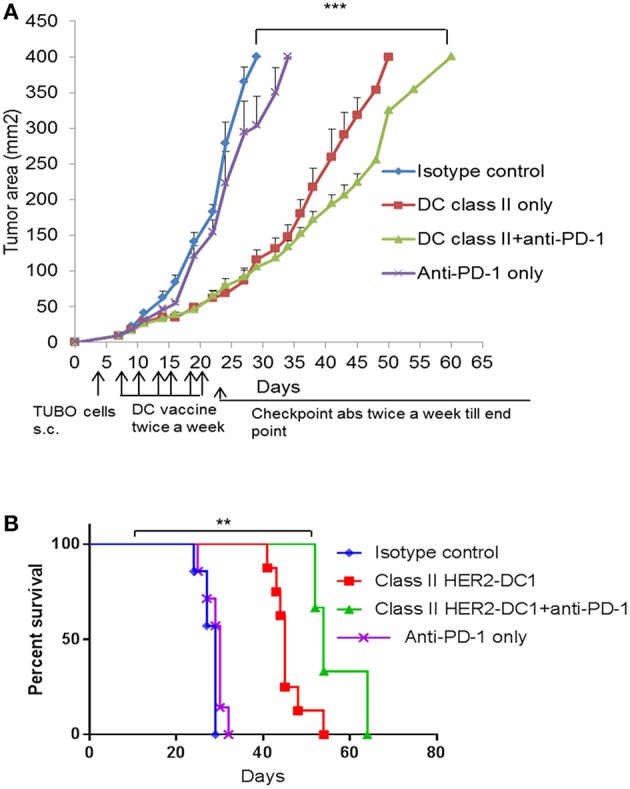
Anti-tumor efficacy of class II HER2 peptides pulsed DC1 in combination with anti-PD-1 antibody therapy. **(A)** Balb/C mice were injected with 2.5 × 10^5^ TUBO cells subcutaneously on the right flank on day 0. When tumors were palpable on day 7, mice received class II HER2 peptides pulsed DC1 vaccine (1 × 10^6^ DC1/mouse/100 μl) subcutaneously on the left flank twice a week for a total of six doses followed by intraperitoneal injection of 150 μg/mouse/200 μl of anti-PD-1 antibody twice a week until the end point. **(B)** Survival curve. Data shown are representative from three independent experiments and are shown as the mean number ± SEM. *P*-values were determined by unpaired student *t-*test **(A)** or a log-rank test **(B)**. ***p < 0.001, **p < 0.01.

The role of CD8^+^ T cells in improving immune checkpoint blockade has been shown previously (39, 40). However, to the best of our knowledge, the role of CD4^+^ helper T cells in facilitating and mediating anti-tumor immune responses in combination with checkpoint blockade has not been studied. To address this, we evaluated therapeutic efficacy of class II HER2-DC1 vaccine in combination with anti-PD-1 antibody therapy. Balb/C mice were injected with TUBO cells on day 0. On day 7, when tumors were palpable, mice received class II HER2-DC1 twice a week for 3 weeks followed by anti-PD-1 antibody therapy twice a week until the end point. Addition of anti-PD1 antibody delayed tumor growth in class II HER2-DC1 vaccinated mice compared to HER2-DC1 alone with survival rate tripled (Figures 6A,B). We also evaluated anti-tumor efficacy of class II HER2-DC1 vaccine in combination with anti-PD-L1 antibodies. We did not observe any additional therapeutic benefit of class II HER2-DC1 vaccine when combined with anti-PD-L1 antibody (Data not shown). These results suggest that checkpoint inhibitors given concurrently with HER2- DC1 vaccine do not have any additive benefit, while administration of anti-PD-1 antibody following generation of anti-HER2 Th1 immune response has an impact on both tumor growth and survival. Overall, these findings suggest that optimal scheduling of immune checkpoints is critical in enhancing the efficacy of HER2-DC1 vaccine.

### Class I and Class II HER2-DC1 Vaccine in Combination With Anti-PD-1 Antibody Therapy Improves T Cell Infiltration, Function, and Specificity

**Figure 7:**
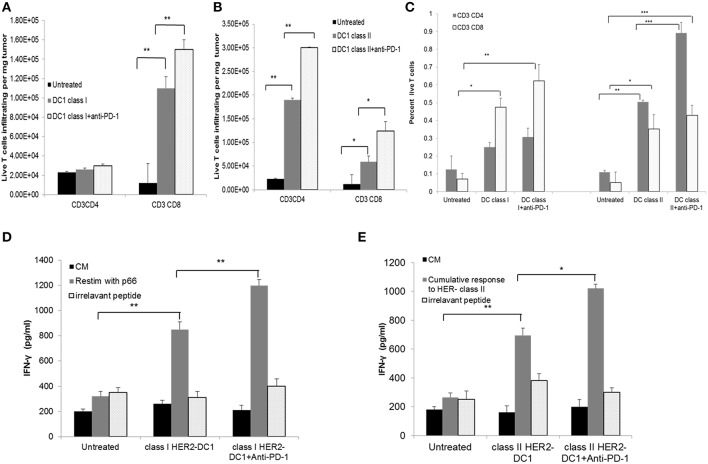
HER2-DC1 vaccine in combination with anti-PD-1 antibody therapy improves T cell infiltration, function, and specificity. **(A,B)** Tumors were collected and single cell suspensions were prepared and stained for CD3, CD4, and CD8 as described in methods. Bar graph shows T cell infiltration per mg of tumor. **(C)** Percent T cells of all live cells within the tumor single cell suspension. **(D,E)** Splenocytes were re-stimulated with class I, class II HER2 peptides or irrelevant OT-I or OT-II peptides. Culture supernatants were collected after 72 h and IFN-γ was measured by standard ELISA. *P*-values were determined by unpaired student *t*-test ***p < 0.001, **p < 0.01, *p < 0.05.

The effect of PD-1 blockade in combination with HER2-DC1 on T cell infiltration, function and specificity was examined. Balb/C mice were injected with TUBO cells on day 0. On day 7, when tumors were palpable, mice received class I or class II HER2-DC1 twice a week for 3 weeks followed by anti-PD-1 antibody therapy. Spleens and tumors were collected from experimental mice on day 35 to examine the T cell infiltration, function and antigen specificity as described in Materials and Methods section and figure legends. Administration of anti-PD-1 antibody in combination with class I HER2-DC1 vaccination increased CD8+ T cell infiltration in tumors (per milligram of tumor) compared to tumor bearing mice that received class I HER2-DC1 only (Figure 7A, *p* < 0.01). We did not observe any changes in CD4+ T cell infiltration per milligram of tumor in mice that received class I HER2-DC1 alone or in combination with anti-PD-1 antibody therapy. Administration of class II HER2-DC1 in combination with anti-PD-1 antibody therapy significantly increased both CD4 and CD8^+^ T cell infiltration per milligram of tumor (*p* < 0.01) compared to HER2-DC1 group alone and untreated controls as shown in Figure 7B. Data shown in Figure 7C represent the percent T cells of all live cells from tumor digest suspension.

Splenocytes from mice that received treatments were re-stimulated with p66 class I HER2 peptide. Irrelevant OVA peptides served as negative controls and did not show any non-specific reactivity. Higher levels of IFN-γ production were observed in class I HER2-DC1 in combination with anti-PD-1 therapy compared to class I HER2-DC1 alone group (Figure 7D, *p* < 0.01). Similar results were observed in mice that received class II HER2-DC1 vaccine when re-stimulated with relevant peptides. Cumulative response to p5, p1209, and p435 peptides is shown in Figure 7E. There was a slight trend toward increased IFN-γ levels between the class II HER2-DC1 alone group and the combination of class II HER2-DC1 and anti-PD-1 antibody treated group in response to re-stimulation with peptides. This data suggest that addition of anti-PD-1 antibody therapy with HER2-DC1 vaccine significantly increases T cell infiltration, enhances tumor specificity and function.

### Effect of Combination Therapy With Class II HER2-DC1 and PD-1 Blockade Is Mediated by CD4^+^ T Cells

**Figure 8:**
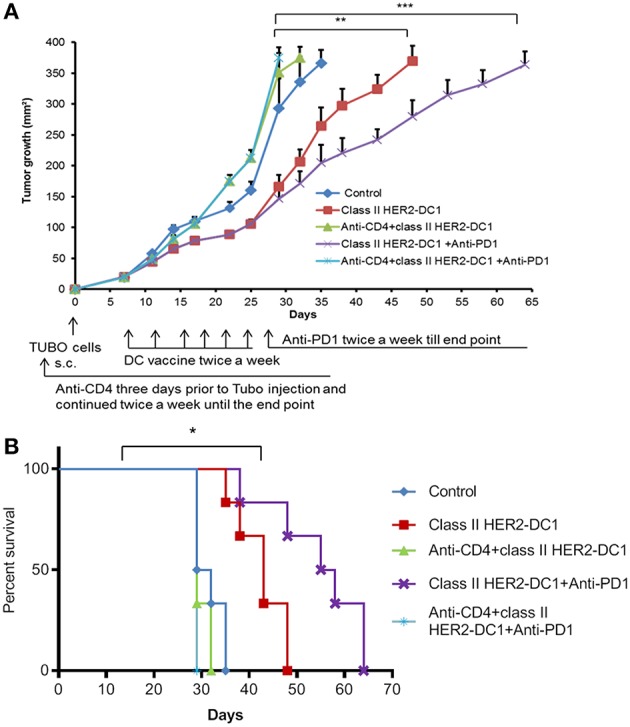
Depletion of CD4+ T cells abrogates the anti-tumor efficacy of HER2-DC1 vaccine and anti-PD1 antibody therapy. **(A)** Balb/c mice were injected s.c. with TUBO cells and treated with class II HER2-DC1 vaccine (1 × 10^6^ DC1/mouse/100 μl, twice a week for 3 weeks) with or without anti-PD1 antibody (150 mg/200 ml/mouse/intraperitoneally twice a week until the end point). CD4 depleting antibody (clone GK1.5) was administered 3 days prior to TUBO induction and continued twice a week until the end point. Tumor growth was monitored periodically. **(B)** Survival curve. Data shown are representative from three independent experiments and are shown as the mean number ± SEM. *P*-values were determined by unpaired student *t-*test **(A)** or a log-rank test **(B)**. ***p < 0.001, **p < 0.01, *p < 0.05.

Our findings suggest that TUBO bearing mice treated with class II HER2-DC1 vaccine can drive both CD4 and CD8^+^ T cell infiltration. To determine the role of CD4^+^ T cells in mediating tumor delay in TUBO tumors, mice were treated with CD4 depleting antibody on day −3 followed by injection of TUBO cells on day 0. When tumors were palpable, mice received either class II HER2-DC1 alone for 6 weeks or followed by anti-PD-1 antibody twice a week until the end point. Mice that received combination treatment with class II HER2-DC1 and anti-PD-1 antibodies in the absence of CD4 T cell depleting antibodies served as positive control. Control mice received isotype antibody control. As shown in Figure 8A, treatment with combination class II HER2-DC1 and anti-PD-1 antibody significantly delayed tumor growth compared to single treatments alone (*p* < 0.03) while combination therapy of class II HER2-DC1 with anti-PD-1 antibody further delayed tumor growth and survival (Figures 8A,B; *p* < 0.002). Depletion of CD4^+^ T cells led not only to the loss of anti-tumor effects mediated by the combination therapy with class II HER2-DC1 and anti-PD-1 but also to more rapid tumor growth and diminished survival. Nevertheless, class II HER2-DC1 vaccine failed to delay tumor growth in the absence of CD4^+^ T cells. Similar results were also observed when class II HER2-DC1 vaccine was combined with anti-PD1 antibody in the absence of CD4^+^ T cells (Figure 8A). This data strongly support a critical role of class II HER2-DC1 vaccine in mediating CD4^+^ Th1 immune responses.

### Synergistic Effect of HER2 Targeted Therapy in Combination With Class II HER2-DC1 Vaccine and PD-1 Blockade Delays Tumor Growth and Enhance Survival Rate

**Figure 9:**
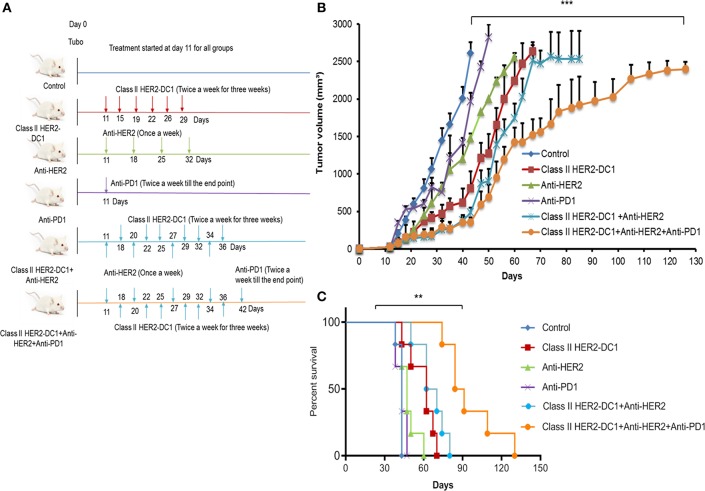
HER2 targeted therapy in combination with HER2-DC1 vaccine and anti-PD-1 therapy delays tumor growth and improves survival. **(A)** The treatment schema. **(B)** Balb/C mice were injected with 3 × 10^4^ TUBO cells into the mammary fat pad to create orthotopic primary tumors. TUBO bearing mice with orthotopic primary tumor were treated with class II HER2 peptide pulsed DC1 vaccine (1 × 10^6^ DC1/mouse/100 μl, twice a week for 3 weeks), anti-PD-1 antibody (two times per week) or the combination of HER2 targeted monoclonal antibodies 7.16.4 and 7.6.5 (once in a week) and class II HER2 peptide pulsed DC1 vaccine followed up with anti-PD-1 antibody twice a week. **(C)** Survival curve. Data shown are representative from three independent experiments and are shown as the mean number ± SEM. *P*-values were determined by unpaired student *t*-test **(B)** or a log-rank test **(C)**. ***p < 0.001, **p < 0.01.

In a PANACEA trial, Pembrolizumab plus trastuzumab showed a clinical benefit in patients with PD-L1-positive, trastuzumab-resistant, advanced, HER2^+^ breast cancer, and in patients with increased TIL. However, the trial did not show any clinical benefit in the PD-L1 negative cohort (18). This trial suggests the importance of immune mechanism in trastuzumab resistance populations, PD-L1 expression on tumors, and TIL infiltration. Since we observed increased TIL infiltration following HER-2 DC1 vaccination and higher levels of PD-1 expression on TIL, we investigated whether combination with anti-HER2 antibodies, class II HER2-DC1 vaccine, and anti-PD-1 therapy have an impact on tumor burden in the TUBO breast cancer model. TUBO cells (30,000 cells) were injected in the mammary fat pad on day 0 and when tumors were palpable, mice received anti-HER2 antibody once a week for 3 weeks or in combination with class II HER2-DC1 given twice a week for 3 weeks. Upon completion of combination treatment, anti-PD-1 antibody was given twice a week until the end point as described in Materials and Methods section. The treatment schema is outlined in Figure 9A. As shown in Figure 9B, blockade of HER2 overexpression using anti-rat neu antibodies (clones 7.16.4 and 7.9.5) significantly reduced tumor burden when combined with class II HER2-DC1 and anti-PD-1 antibody (Figure 9; *p* < 0.001). The class II HER2-DC1 vaccine treatment alone significantly delayed tumor growth compared to TUBO bearing control mice and doubled the survival rate. We observed no significant effect in delaying tumor growth in TUBO bearing mice treated with anti-PD-1 antibody alone while anti-rat neu antibody had minimal effect in delaying tumor growth. The combination of class II HER2-DC1 alone with anti-rat neu antibodies 7.16.4 and 7.6.5 did not have any significant delay in tumor growth compared to class II HER2-DC1 alone. Interestingly, the addition of anti-PD-1 antibody with monoclonal anti-rat neu antibodies 7.16.4 and 7.6.5 and class II HER2-DC1 vaccine not only significantly delayed the tumor growth but also enhanced and quadrupled the survival rate from control mice (Figures 9B,C). This data suggest that addition of HER2 targeted therapy to the combination of HER2-DC1 vaccine and anti-PD-1 antibody reduces tumor burden and improves survival rate in an orthotopic HER2 positive breast cancer model.

## Discussion

HER2 overexpression/amplification accounts for about 25% of breast cancers and is associated with aggressive disease and poor clinical prognosis. As previously discussed, the humanized monoclonal antibodies (trastuzumab and pertuzumab) directed against HER2 in combination with chemotherapy have been demonstrated to be clinically effective for the treatment of patients with HER2^+^ MBC (41). Other therapeutic options for these patients include combinations of Lapatinib (an oral tyrosine kinase inhibitor), T-DM1, and chemotherapies (42). It should be emphasized however that patients with HER2^+^ MBC will eventually face disease progression while receiving treatment with currently approved HER2-targeted therapies. Development of new treatments is an obvious unmet clinical need not only for patients with *de novo* MBC but also for the subset of patients with HER2^+^ resectable breast cancer who will have MBC recurrence despite multimodality treatment (43, 44). Therefore, effective therapy to overcome resistance and improve the clinical response in metastatic patients is warranted.

In this study, for the first time we describe the anti-tumor efficacy of MHC class I HER2 peptide p66 and class II HER2 peptides (p5, p435, and p1209) pulsed DC1 vaccine alone or in combination with immune checkpoint blockade and HER2-targeted therapy in a preclinical model of HER2 over expressing breast cancer. Vaccination with class I HER2-DC1 or class II HER2-DC1 generated anti-HER2 Th1 immune responses and delayed tumor growth. In contrast, there was no enhanced anti-tumor efficacy in mice vaccinated with both class I and class II HER2 peptide pulsed DC1 compared to mice vaccinated with class I or class II HER2-DC1 alone. While class I HER2-DC1 enhanced only CD8^+^ T cell infiltration but not CD4^+^ T cells, class II HER2-DC1 vaccination induced both CD4 and CD8^+^ T cell infiltration, suggesting that vaccination with class II HER2-DC1 may be sufficient in generating anti-tumor immunity and HER2-specific immune responses. Re-stimulation of splenocytes from vaccinated mice with class II HER2 peptides led to increased IFN-γ production, suggesting the critical role of the anti-HER2 CD4^+^ Th1 immune response in mediating reduction in tumor burden with enhanced survival benefit. IFN-γ is recognized as a key cytokine in mediating CD4^+^ and CD8^+^ Th1 immune responses (45, 46). We have previously shown that TLR-4 activated DC1 inhibit CD4^+^CD25^+^FoxP3^+^ regulatory T cells and converts them in to Th1 like effector cells. These Th1 like effector cells co-expressed T-bet and increased IFN-γ production. This suggests the critical role of DC1 in mediating CD4^+^ Th1 immune responses (47). A recent study from our lab reported that IFN-γ eliminates HER2 expressing breast cancer cells through JAK-STAT-1 dependent induction of senescence and apoptosis (48). Our preclinical findings corroborate the clinical trial data showing that treatment of HER2 positive invasive breast cancer patients with HER2 peptide-pulsed DC1 vaccine resulted in successful restoration of anti-HER2 Th1 immune response with improved pCR (28, 31, 49).

Various clinical findings have shown that HER2 expressing breast cancer cells in the tumor microenvironment utilize the PD-1/PD-L1 dominant immune checkpoint pathway to down regulate anti-tumor immune cells function and evade immune cells mediated tumor eradication (50, 51). Importantly, PD-L1, a ligand for PD-1 is constitutively expressed on HER2 overexpressing breast cancer (52). Despite the reports that monoclonal antibodies that directly inhibit PD-1 and PD-L1 have been a successful treatment options for advanced melanoma, non-small cell lung carcinoma (NSCLC), and some patients with high mutational burden like HNPCC colorectal cancer, limited success rate was noted in breast cancer clinical trials (53). The combination of trastuzumab and pembrolizumab in HER2 resistant advanced breast cancer patients had modest clinical benefit only in the PD-L1 positive cohort (18) or in those with at least some TIL infiltration and no benefit was seen in the PD-L1 negative cohort.

Our preclinical findings suggest that both class I and class II HER2-DC1 increased infiltration of TIL which express high levels of PD-1 receptor. Class I HER2-DC1 vaccine given sequentially in combination with anti-PD-1 antibody therapy had an impact in reducing tumor burden with improved survival benefit. In contrast, HER2-DC1 vaccine and anti-PD-1 antibody given concurrently did not generate any synergistic effect. These results corroborate with a recent study indicating that in MMTV-PyMT mammary cancer model, concurrent treatment with anti-PD1 antibody and anti-OX-40 diminished the therapeutic efficacy. However, sequential treatment with anti-PD-1 and anti-OX-40 resulted in improved therapeutic efficacy and was associated with worse outcomes and increased T cell apoptosis (54). This supports our data suggesting that sequence and timing of checkpoint blockade is critical for combinatorial strategies. The optimal impact of checkpoint antibody treatment on tumor growth and resistance also likely depends in part on the tumor burden. A recent study demonstrated the anti-tumor efficacy of anti-CTLA-4 and anti-PD-1 in the low tumor burden state in pre-clinical melanoma model as well as in melanoma patients. Their data suggest that in the low tumor burden setting, combination therapy induced higher levels of IFN-γ receptor on activated tumor specific T cells which were more susceptible to apoptosis than naïve T cells. In this setting, combination therapy induced deletion of tumor-specific T cells and altered the T cell repertoire compared to the high tumor burden setting. The authors suggest that there is a less exhausted immune status in the low tumor burden state. In addition, they suggest that in the setting of high tumor burden, duration of antigen exposure and antigen loads could alter or reprogram the exhaustion status of T cell profile (55). We believe that in our model, one possible reason for the difference in response to checkpoint therapy could be that mice receiving concurrent HER2-DC1 and anti-PD-1 antibody treatment in the low tumor burden setting made anti-PD-1 therapy less effective. While in the mice receiving sequential treatment, where anti-PD-1 therapy was given in a setting of higher tumor burden, treatment was more effective. Altogether our results indicate that the effect of checkpoint blockade largely depends on optimal scheduling to be successful with other immunotherapeutic strategies.

Class II HER2-DC1, when combined with anti-PD-1 antibody, quadrupled the survival rate with increased anti-HER2 CD4^+^ Th1 immune response and increased CD4^+^ and CD8^+^ T cells infiltration within the tumor. Interestingly, depletion of CD4^+^ T cells completely abrogated the anti-tumor efficacy of the class II HER2-DC1 alone or in combination with anti-PD-1 therapy suggesting the crucial role of CD4^+^ T cells. A recent study indicates that NSCLC patients with highly dysfunctional CD4 immunity had no objective response to PD-1/PD-L1 blockade therapy. However, in patients with non-dysfunctional CD4 responses there was a response rate of about 50% to PD-1/PD-L1 blockade therapy. More importantly, CD8 immunity was recovered only in patients with functional CD4 immunity (56). These data support our findings that boosting the anti-HER2 CD4^+^ Th1 immune responses prior to immune checkpoint blockade will be beneficial in breast cancer patients.

In contrast, no effect on delaying tumor growth was observed when class I or class II HER2-DC1 vaccine was combined with anti-PD-L1 antibody. In the clinical setting, Avelumab, a PD-L1- antibody, has been shown to have only modest responses in breast cancer subtypes in the JAVELIN study (57). It is well-known that IFN-γ upregulates PD-L1 expression (45). Despite the fact that HER2^+^ TUBO cells expressed higher levels of PD-L1, we were unable to observe synergy with anti-PD-L1 antibody and HER2-DC1 vaccine. Further studies are warranted on the optimal dosing and scheduling of anti-PD-L1 antibody and to validate its efficacy in combination with HER2-DC1 vaccine.

Our data suggests that addition of anti-HER2 antibodies further enhanced the efficacy of class II HER2-DC1 vaccine in combination with anti-PD-1 antibody, with a prolonged survival advantage. In a HER2 overexpressing and trastuzumab resistant preclinical breast cancer model, targeted treatment with ado-trastuzumab emtansine (T-DM1) in combination with anti-PD1 antibody or anti-CTLA-4 antibody showed enhanced anti-tumor efficacy, T cells trafficking in to the tumor and Th1 cell polarization (58). Preclinical studies combining IFN-γ and anti-HER2 antibody have been shown to induce a synergistic effect in reducing HER2 expressing orthotopic mammary tumor growth *in vivo* (14). Taken together, this study highlights the critical role of CD4^+^ T cells and the use of class II HER2-DC1 vaccine in combination with immune checkpoint blockade and HER2 targeted therapy in facilitating and mediating anti-HER2 Th1 immune responses. This combinatorial approach could be directly translated to clinical settings.

## Data Availability

The datasets generated for this study are available on request to the corresponding author.

## Author Contributions

KK and BC conceived and designed the experiments and supervised the work. KK, CS, GR, AB, YJ, HZ, and LL performed the experiments. KK, CS, GR, and BC analyzed data and contributed to data analysis. KK, BC, and RC wrote the manuscript. GR, CS, DW, APB, SA, AB, LL, HZ, MG, RC, and YJ edited the manuscript.

### Conflict of Interest Statement

The authors declare that the research was conducted in the absence of any commercial or financial relationships that could be construed as a potential conflict of interest.

## Abbreviations

BM: Bone marrow
BSA: bovine serum albumin
Class I HER2-DC1: Class I HER2 pulsed DC1 vaccine
Class II HER2-DC1: Class II HER2 peptide pulsed DC1
DAPI: 4′6-diamindino-2-phenylindole
DC1: Type I polarized dendritic cells
FMO: Fluorescent minus one
iDC: Immature DC
LPS: lipopolysaccharide
MBC: Metastatic HER2 breast cancer
mOS: Median overall survival
NSCLC: Non- small cell lung carcinoma
PD-1: Programmed death 1 receptor
PD-L1: Programmed cell death 1 ligand 1
PVDF: Polyvinylidene difluoride
rHER2: rat HER2 oncogene/peptide
s.c.: Subcutaneously
T-DM1: Ado-trastuzumab/ado-trastuzumab emtansine
Th1: T helper cell
TIL: Tumor infiltrating lymphocyte.
